# THE STUDY OF AGING A SEARCH FOR MEANINGFUL PATIENT AND PUBLIC INVOLVEMENT IN GERONTOLOGICAL QUALITATIVE DATA ANALYSIS

**DOI:** 10.1093/geroni/igz038.2953

**Published:** 2019-11-08

**Authors:** Barbara Hanratty, Rachel Stocker, Katie Brittain

**Affiliations:** 1 Newcastle University, Newcastle upon Tyne, United Kingdom; 2 Northumbria University, Newcastle, United Kingdom

## Abstract

Patients and the public are involved in health and social care research more than ever before. Much effort has been put into developing patient and public involvement (PPI), and promoting co-production of research with patients and the public. Yet there is little guidance for researchers on how to involve PPI partners in the research process, or how involvement can be judged as meaningful. This presentation has its origins in the attempts of one research team to question and navigate a way of involving PPI in long term care research. In this presentation, we describe our model of collaborative qualitative data analysis with PPI partners, in a study exploring primary care services for older adults living in long-term care facilities in England. Anonymised interview transcript excerpts were presented in written, audio, and role-play format to our PPI partners. PPI partners derived meaning from interview data, identifying, confirming and critiquing emerging themes. Their input at this critical stage of the study deepened our initial analysis and prompted the research team to new and different interpretations of the data. This talk addresses ways of engaging PPI partners in innovative ways during data analysis, and offers other researchers some questions, challenges and potential principles for effective practice. We conclude that in areas such as long term care, with multiple stakeholders and a dynamic environment, effective PPI may be flexible, messy and difficult to define.

